# Evaluation of the aMAP score for hepatocellular carcinoma surveillance: a realistic opportunity to risk stratify

**DOI:** 10.1038/s41416-022-01851-1

**Published:** 2022-07-07

**Authors:** Philip J. Johnson, Hamish Innes, David M. Hughes, Anton Kalyuzhnyy, Takashi Kumada, Hidenori Toyoda

**Affiliations:** 1 Department of Molecular and Clinical Cancer Medicine, University of Liverpool, Liverpool, UK; 2 School of Health and Life Sciences, Glasgow Caledonian University, Glasgow, UK; 3 Division of Epidemiology and Public Health, University of Nottingham, Nottingham, UK; 4 Public Health Scotland, Glasgow, UK; 5 Department of Health Data Science, University of Liverpool, Liverpool, UK; 6 Computational Biology Facility, University of Liverpool, Liverpool, UK; 7 Department of Nursing, Gifu Kyoritsu University, Ogaki, Japan; 8 Department of Gastroenterology, Ogaki Municipal Hospital, Ogaki, Japan

**Keywords:** Predictive markers, Hepatocellular carcinoma

## Abstract

**Background and aims:**

The aMAP score is a model that predicts risk of hepatocellular carcinoma (HCC) development in patients with chronic hepatitis. Its performance in a ‘real world’ surveillance setting has not yet been ascertained.

**Patients and methods:**

We had access to a cohort of 3473 individuals enrolled in a rigorously implemented and prospectively accrued surveillance programme (patients undergoing regular ultrasound and biomarker examination between 1998 and 2021). During this period 445 had HCC detected. Of these, 77.8% had early stage disease (within Milan criteria), permitting potentially curative therapy to be implemented in nearly 70% of cases. We applied the recently developed aMAP score to classify patients according to their initial aMAP score in to low, medium and high-risk groups as proposed in the original publication. The performance of the aMAP score was assessed according to the concordance-index and calibration (i.e. agreement between observed and predicted risk). Allowance was made for competing causes of death.

**Results:**

The aMAP score achieved an overall *C*-index of 0.81 (95% CI: 0.79–0.82) consistent with the initial report and was unaffected by allowance for competing causes of death. Sub-group analysis showed that the results did not change significantly according to gender, or aetiology. However, aMAP discrimination was greater for younger individuals (versus older individuals), and also for individuals without cirrhosis. The HCC incidence rate was 0.98, 7.05 and 29.1 events per 1000 person-years in the low-, moderate- and high-risk aMAP groups, respectively.

**Conclusions:**

The results from this ‘real-world’ cohort demonstrate that risk stratification is a realistic prospect and that identification of a subgroup of chronic liver disease patients who have a very low risk of HCC is feasible.

## Introduction

Potentially curative therapies for hepatocellular carcinoma (HCC) rely on early diagnosis [1]. To achieve this outcome regular, typically six-monthly, ultrasound (US) examinations of patients with hepatic cirrhosis is recommended, with or without the tumour marker alpha-fetoprotein (AFP) [2]. However, uptake into surveillance programmes has been poor particularly across the western world where only 20–40% of patients developing HCC have been detected within a surveillance programme and early diagnosis remains uncommon [3]. The barriers to effective implementation have been clearly identified [4] and one of these is that not all clinical or funding agencies have been convinced of the value of surveillance since there has been no acceptable controlled study [5]. Harms have been identified, not least of which has been the anxiety engendered by regular scans, each of which may reveal a potentially lethal cancer and ‘false-positive’ results [6].

In Japan, a government-funded and well-designed surveillance programme which was started in the 1980s, probably represents the upper limit of what can be expected from current surveillance strategies [7]. Median survival of those detected with HCC has increased, decade on decade, from <4 months in the 1980s (as it was then in other countries) to >4 years [7, 8] currently. Since 2000 such advances seem to reflect improving underlying liver function as well as earlier detection and better treatment [9, 10]. The recently developed aMAP score, based on age, gender, platelet count and the ALBI score [11], appears to accurately predict the likelihood of HCC development in populations under surveillance, paving the way for practical risk stratification strategies [12]. Notably, recognising the difficulty of accurate and consistent diagnosis of ‘cirrhosis’ [13], the model was developed in patients with chronic hepatitis without specific reference to the presence or absence of cirrhosis. However, clinical application of such scores conventionally requires long-term validation studies in a setting similar to that in which the test would be applied.

We have access to data from a routine but rigorous surveillance programme in a district general hospital in Japan, in which all relevant clinical and laboratory features have been prospectively recorded over more than 20 years. Specifically, the parameters necessary to calculate the aMAP score were recorded prior to the development of the outcome which the score aims to predict, namely HCC. We have previously undertaken detailed analysis of this programme showing that survival is significantly better than in similar communities in Hong Kong and the UK, where surveillance programmes are not strictly implemented, even allowing for lead-time bias [14]. This permits us, in effect, to judge the efficacy of the aMAP score in a robust manner thereby giving the opportunity to decrease the period between method (in this case, the aMAP score), discovery and practical implementation.

## Patients and methods

### The Ogaki cohort

The present study included all patients without prior HCC, who were recruited to the HCC surveillance programme at the Ogaki Municipal Hospital between March 1998 and April 2014 and followed up until 2021. Ogaki Municipal Hospital is a general hospital serving a well-defined and stable local population of approximately 400,000. Of all HCC patients seen in this municipality, more than 70% were detected under surveillance [15]. Data on all incident HCCs and mortality events occurring in this cohort were available through to March 2021. The diagnosis of HCC was made according to the European Association for the Study of the Liver guidelines but the high incidence of resection meant that over 50% were confirmed histologically. AFP levels greater than 20 ng/mL or a positive US triggered a diagnostic workup for HCC with computed tomography or magnetic resonance imaging. The diagnosis of chronic liver disease (CLD)/cirrhosis was established at the time of enrolment into the surveillance programme by experienced clinicians using clinical, laboratory and radiological features. Henceforth, the presence or absence of cirrhosis refers to status on entry into the study although as noted below some of those in the ‘no-cirrhosis’ category did progress to cirrhosis during the follow-up period.

The aMAP score was calculated for each patient by applying the original formula [12]:

aMAP risk score = ({0.06 × age + 0.89 × sex (Male: 1, Female: 0)

+ 0.48 × [(log_10_ bilirubin × 0.66)

+ (albumin × −0.085)] − 0.01

× platelets} + 7.4)/14.77 × 100

### Statistical analysis

All statistical analyses were underpinned by survival analysis methods. Follow-up time began at the date of the first available aMAP score following entry into the HCC surveillance programme. This was defined as the first date at which the laboratory components of the aMAP score—i.e. platelet count, albumin and bilirubin—were measured simultaneously (i.e. from the same blood specimen). Follow-up time ended at the date of definitive diagnoses of HCC (if at all), mortality (if at all) or the date of last clinic visit. Patients were defined as lost to follow-up if they were not known to have died by December 2020, and if they had not had a clinic visit in more than 12 months (i.e. last clinic visit prior to December 2019).

As recommended by best-practice guidelines [16], the performance of the aMAP model was evaluated in terms of:

- Discriminative ability (i.e. ability of aMAP to differentiate patients who develop HCC and those who do not) and
- Calibration (i.e. agreement between the 5-year probability of HCC predicted by aMAP and the 5-year probability of HCC observed in the Ogaki cohort).

Discriminative ability of aMAP was first assessed visually, by plotting the Kaplan–Meier estimate of the survivor function for the low-, moderate- and high-risk aMAP groups. The cut off points for these three groups were based on those proposed in the original paper (i.e. <50 = low risk; 50–59 = moderate risk; >60 = high risk). Secondly, we calculated the discriminative ability quantitatively using the concordance index (CI). In our base-case analysis, we used the standard Harrell’s *C*-index; however, we also calculated the Wolbers-modified version of the *C*-index assessing the impact of non-HCC mortality as a competing risk event. For all versions of the *C*-index, higher values indicate better discrimination. A *C*-index of 0.50 indicates zero discrimination (i.e. no better than chance), whereas a *C*-index of 1.0 indicates perfect discrimination.

Further, we assessed if the C-index of each prediction model varied according to selected patient characteristics. The characteristics were as follows: presence of cirrhosis at baseline; gender; age; liver disease aetiology and sustained virological response (SVR) status. Finally, we also calculated the discrimination of aMAP over various time horizons to assess if it performs better over shorter-term time periods versus longer-term time periods. The time horizons we considered were: 0–1, 0–2, 0–3, 0–4, 0–5, and 0–6 years after baseline.

The second aspect of model performance—Calibration—was assessed by comparing the 5-year probability of developing HCC with the observed proportions of individuals developing HCC in our cohort, using deciles of predicted risk. The predicted 5-year probability of aMAP was evaluated using the equation prescribed by the authors of the aMAP score, namely, 1 − *S*_0_(*t*)^exp(linear predictor)^ where *t* = *X* years, and *S*_0_(*t*) refers to the 5-year baseline survival function (*S*_0_(*t*) = 0.984). The 5-year observed HCC probability was based on Kaplan–Meier estimates of cumulative incidence. However, in a sensitivity analysis, this was alternatively defined as the 5-year cumulative incidence of HCC, accounting for non-HCC mortality as a competing risk event. A competing risk perspective ensures that the observed 5-year HCC probability is not overestimated, as can be the case with the standard Kaplan Meier estimate.

### HCC mortality compared to the general population

We calculated the standardised mortality ratio (SMR) for liver cancer mortality according to the aMAP risk group. SMRs represent the ratio of the number of expected deaths to the number of observed deaths. Here the number of expected deaths was based on the number of liver cancer deaths one would expect to observe in the Ogaki cohort if the mortality rate was identical to that seen in the general population of Japan. All SMRs were adjusted for age, sex and calendar year. In this way, differences in the observed and expected number of deaths will be independent of differences in age/sex/calendar period between the Ogaki cohort and the general population. The total number of deaths from liver cancer in Japan, according to age, sex and calendar year was obtained from National Cancer Centre of Japan (https://ganjoho.jp/reg_stat/index.html). Of note, SMRs were calculated for liver cancer mortality as opposed to HCC mortality because data for the latter were unavailable. However, the vast majority (>90%) of liver cancer deaths in Japan are from HCC [17].

## Results

### Characteristics of the sample

A total of 3479 patients met our inclusion criteria (i.e. all patients without prior HCC, who were recruited to the HCC surveillance programme at the Ogaki Municipal Hospital between March 1998 and April 2014). Six patients were excluded due to an unknown aMAP score. Thus, our final sample comprised 3473 patients.

In the final sample, the median age at baseline was 61.0 years with a similar proportion of males (49.4%) and females (50.6%). On entry into the surveillance programme, 55% of the patients in the were hepatitis C virus (HCV) seropositive, 24.1% were seropositive for hepatitis B virus and 21.3% had other forms of CLD (Table 1). Less than 1% of patients with HCV aetiology had achieved a sustained viral response at baseline. In contrast, by the end of follow-up, more than half of all HCV patients had achieved SVR (1026/1912; 53.7%)—mainly following direct-acting antiviral (DAA) regimens.

**Table 1:** Demographics, clinical and laboratory features of the cohort at baseline.

Variable	Group		
	All (*n*)	No HCC	HCC
Patient count	3473 (100%)	3028 (100%)	445 (100%)
Age at assay day (years) (Median and range)	61.0 8.9 to 90.3	60.3 8.9 to 90.3	65.0 27.3 to 85.6
Male, *n* (% of group)	1715 (49.4%)	1446 (47.8%)	269 (60.4%)
Female, *n* (% of group)	1758 (50.6%)	1582 (52.2%)	176 (39.6%)
Aetiology, *n* (% of group):			
HCV	1912 (55.0%)	1563 (51.6%)	349 (78.4%)
HBV	837 (24.1%)	773 (25.5%)	64 (14.3%)
Other	740 (21.3%)	703 (23.2%)	37 (8.3%)
Liver cirrhosis, *n* (% of group)	886 (25.5%)	612 (20.2%)	274 (61.6%)
ALBI grade, *n* (% of group):			
Grade 1	2660 (76.6%)	2443 (80.7%)	217 (48.8%)
Grade 2	758 (21.8%)	546 (18.0%)	212 (47.6%)
Grade 3	55 (1.6%)	39 (1.3%)	16 (3.6%)
ALBI score (median and range)	−2.87 −3.79 to −0.17	−2.91 −3.79 to −0.17	−2.57 −3.62 to −0.51
Albumin (g/l) (median and range)	42 18 to 52	42 18 to 52	39 20 to 50
Bilirubin (µmol/l) (median and range)	10.0 1.7 to 326.7	10.0 1.7 to 208.3	11.7 3.3 to 326.7
AST (IU/l) (median and range)	34 3 to 3412	31 3 to 3412	58 13 to 679
Platelet count (×10^3^/mm^3^) (median and range)	185 8 to 889	193 8 to 889	123 12 to 470

All values relate to the baseline time point (i.e. date of first available aMAP score). Liver cirrhosis status, however, refers to cirrhosis status at the time of enrolment into screening programme.

According to the first recorded aMAP score, approximately one third of the overall cohort fell into each of the three risk categories as defined in the original publication (Table 2)—low risk 27%, medium risk 41% and high risk 32%. However, patients with liver cirrhosis were heavily skewed towards the higher risk aMAP group (Table 2). Furthermore, it is inevitable that the aMAP score will change over time but such dynamic changes within the cohort together with changes in cirrhosis status will be considered in future studies.

**Table 2:** Number of patients according to aMAP group, cirrhosis status and HCC.

aMAP risk group	Liver cirrhosis (*n* = 886)		No cirrhosis (*n* = 2587)		All patients (*n* = 3473)	
	No HCC (*n* = 612)	HCC (*n* = 274)	No HCC (*n* = 2416)	HCC (*n* = 171)	No HCC (*n* = 3028)	HCC (*n* = 445)
Low	8 (1%)	0 (0%)	921 (38%)	12 (7%)	929 (31%)	12 (3%)
Medium	159 (26%)	39 (14%)	1124 (47%)	89 (52%)	1283 (42%)	128 (29%)
High	445 (73%)	235 (86%)	371 (15%)	70 (41%)	816 (27%)	305 (69%)

Numbers in brackets indicate column percentages, rounded up to the nearest whole number.

### Follow-up

The cohort was followed up for a total of 40,825-person years, equating to a mean-average of 11.8 years per patient (range 0.03 to 22.6 years). The median follow-up was 11.4 years, with an inter-quartile range of 7.7 to 15.7 years. There were 829 (24%) patients lost to follow-up at the date of study completion on the basis that they had not attended an appointment in more than 12 months despite not being known to have died (Fig. S1).

Of the 2587 who were classified on clinical grounds as not having cirrhosis at study entry, 187 developed HCC during the course of the study. Of these 187, analysis of serially recorded FIB-4 values suggested that 51% had become cirrhotic (FIB-4 >3.25) by the time they had HCC detected.

### Incident HCC

During 40,825-person years of follow-up, 445 incident HCC events were observed. In the overall cohort, the crude incidence rate for HCC was 10.9 (95% CI: 9.9–12.0) events per 1000-person years follow-up. The crude incidence rate was 0.98, 7.05 and 29.1 events per 1000-person years for individuals in the low, moderate and high risk aMAP groups, respectively. Incidence was also considerably higher for cirrhosis patients (33.7 events per 1000-person years) than non-cirrhosis patients (5.2 events as per 1000-person years).

**Fig. 1:**
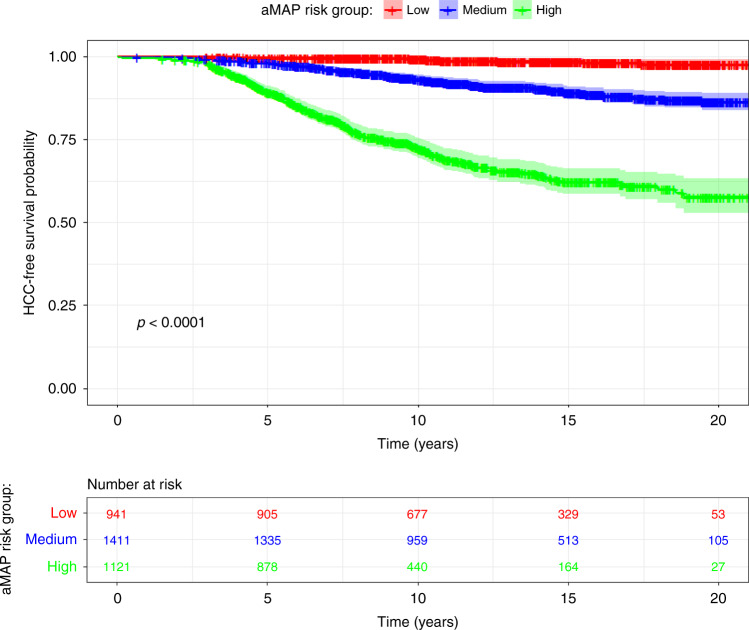
Kaplan–Meier curves showing risk of developing HCC for individuals with low, medium or high risk according to the aMAP score. The *p* value shown is the log-rank test for equality of survival.

Of note of the 2587 patients who were classified, on clinical grounds, as not having cirrhosis on enrolment, 171 patients developed HCC during the course of the study. However, of these 171 patients, analysis of serially recorded FIB-4 values suggested that 54% had become cirrhotic (FIB-4 >3.25) by the time HCC was detected.

The median maximum tumour size at HCC diagnosis was 2 cm, 77.8% were within Milan criteria and 69.4% (309 of 445) underwent treatment with curative intent (Table S1).

### Mortality

The cause of death was recorded in >95% of cases permitting assessment of the impact of competing causes of death on the underlying aMAP assessment. In total, there were 184 deaths from primary liver cancer during follow-up, of which all were from HCC. The crude mortality rate for primary liver cancer mortality was 4.3 events per 1000-person years (95% CI: 3.7–5.0). There were 468 competing risk events (i.e. non-HCC deaths occurring before an HCC diagnosis), equating to a crude incidence of 10.9 events per 1000-person years (95% CI: 10.0–12.0).

### Model performance

#### Discrimination

Kaplan–Meier curves indicated significant differences in HCC incidence for low, moderate and high aMAP groups (*p* < 0.0001; Fig. 1). Overall discrimination was consistent with those reported in the original aMAP publication, with a concordance index of 0.81 [95% CI: 0.79–0.82]. The Wolbers-modified concordance index, accounting for non-HCC mortality as a competing risk, was only marginally lower at 0.78 (95% CI: 0.76–0.80). Discrimination did not vary by liver disease aetiology. However, aMAP discriminated better in younger patients versus older patients, for males versus females, and for cirrhosis patients versus non-cirrhosis patients (See Table 3). For example, *C*-index was 0.87 (95% CI: 0.815, 0.930) for patients aged <51 years versus 0.73 (0.687, 0.763) for patients aged ≥68 years. aMAP also exhibited better discrimination over shorter prediction horizons—i.e. the *C*-index based only on data from the first year of follow-up was 0.88 (95% CI: 0.78–0.98) versus 0.81 (95% CI: 0.79–0.82) for all years of follow-up (Fig. S2). However, *C*-index values relating to the first 1–2 years of follow-up were imprecise, and thus these differences may merely reflect sampling error.

**Table 3:** Subgroup validation of the aMAP risk prediction.

		Number of Individuals	HCC cases	Incidence rate (per 1000 patient years)			*C*-index
				aMAP category			
				Low	Medium	High	
Overall		3473	445	0.98	7.1	29.1	0.81 (0.787, 0.823)
First diagnosis	Chronic hepatitis	2587	171	0.99	5.6	14.9	0.76 (0.728, 0.793)
	Liver cirrhosis	886	274	0	17.3	40.9	0.63 (0.601, 0.668)
Sex	Male	1715	269	1.4	6.8	27.6	0.77 (0.743, 0.796)
	Female	1758	176	0.8	7.3	33.1	0.84 (0.819, 0.867)
Age	<51	819	37	1.0	6.6	42.7	0.87 (0.815, 0.930)
	51–61	914	116	1.3	7.8	32.8	0.80 (0.763, 0.840)
	61–68	818	138	0	7.2	29.0	0.76 (0.720, 0.797)
	≥68	922	154	0	5.5	27.3	0.73 (0.687, 0.763)
Aetiology	Hepatitis B	821	59	1.5	4.2	25.5	0.81 (0.759, 0.865)
	Hepatitis C	1896	344	1.0	9.2	34.1	0.78 (0.760, 0.802)
	Other	756	42	0	3.5	14.9	0.83 (0.784, 0.885)
Achieved SVR^a^	DAAs or interferon	1026	14	0	2.0	9.9	0.81 (0.720, 0.890)
ALBI grade	1	2660	217	0.7	6.1	18.6	0.78 (0.752, 0.805)
	>1	813	228	4.2	13.6	43.9	0.69 (0.658, 0.724)

Incidence rates per 1000 patient years are reported for low, medium and high risk based on aMAP. *C*-index of the aMAP score is also reported.

^a^Of whom 728 had known aMAP status after achievement of SVR.

#### Calibration

**Fig. 2:**
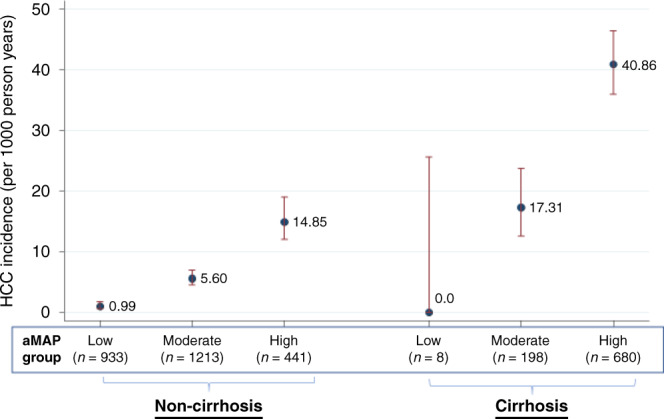
HCC incidence by cirrhosis status and aMAP group.

The model predicted 93.3% of patients to survive 5 years without developing HCC, while we observed 5-year HCC free survival in 95.8% of patients. The aMAP score tended to over-predict the 5-year risk of HCC slightly in our cohort, as shown by the calibration plot in Fig. 2. The over-prediction appears to be worse at higher predicted risks of HCC. For example, in the highest risk decile, the mean 5-year HCC probability predicted by aMAP was 26.8%, whereas the observed 5-year Kaplan–Meier failure estimate was 16.5% (95% CI: 12.3–20.4%). A very similar picture was evident when the 5-year observed probability was defined in terms of the cumulative incidence of HCC accounting for non-HCC mortality as a competing risk event (see Fig. S3).

#### Standardised mortality ratios

The number of deaths from liver cancer was 8.0 times higher than the general population (SMR: 8.0; 95% CI: 7.0–9.3). However, this varied from SMR:1.6 (95% CI: 0.4–6.3) in the low-risk aMAP group to SMR: 11.5 (95% CI: 9.7–13.5) in high-risk aMAP patients.

For patients with cirrhosis, the number of liver deaths was 17.2 times higher than the general population (SMR: 17.2: 95% CI: 14.6–21.3) (see Table 4).

**Table 4:** Liver cancer standardised mortality ratio (SMR0 for the Ogaki cohort relative to the general population of Japan.

Risk group	Observed	Expected	SMR (95% CI)
Full cohort			
All patients	184	22.9	8.0 (7.0–9.3)
aMAP ‘low risk’	2	1.3	1.6 (0.4–6.3)
aMAP ‘mod risk’	41	9.3	4.4 (3.2–6.0)
aMAP ‘high risk’	141	12.3	11.5 (9.7–13.5)
Cirrhosis patients			
All patients	120	7.0	17.2 (14.3–20.5)
aMAP ‘low risk’	0	0	NA
aMAP ‘mod risk’	12	0.9	14.0 (8.09–24.6)
aMAP ‘high risk’	108	6.1	17.6 (14.6–21.3)

SMRs are adjusted for age, sex and calendar period. Expected is the number of liver cancer deaths expected in Ogaki cohort if the age/sex/calendar period adjusted mortality rate was equal to the general population.

## Discussion

Although the aMAP score has been validated in the original [12], and subsequent publications [18, 19], and shown to be the best-performing of current HCC prediction models [20], its role has not yet been established in the prospective setting. Strictly speaking the approach adopted here is to test the aMAP algorithm for risk stratification in patients with chronic hepatitis but it cannot claim to be a prospective study insomuch as it was not undertaken explicitly to test aMAP performance in routine clinical surveillance practice. However, the dataset, as well as being completely independent, does overcome some of the limitations noted in the original publication and it has several unique features that render it ideal for model evaluation.

First, although the aMAP score was not a stated objective, all the contributing parameters (variables within the model) were prospectively accrued prior to the detection of the outcome (HCC development) that the score aims to predict. Secondly, it provides important information about the performance of the model in a ‘real-world’ setting. Thirdly, surveillance was applied in a district general hospital (as opposed to a specialist unit) in a rigorous manner conforming to current international guidelines and in a setting with considerable expertise in both ultrasound examination and radiological interpretation. The fact that the model is applicable to all patients with CLD, not simply cirrhosis, is also a strength although models for specific subgroups such as those achieving SVR may still be highly relevant [21]. Current Western guidelines suggest that only patients with established cirrhosis (apart from certain ‘high-risk groups’) should enter surveillance programmes. In this series our analysis implies that rigid confinement to cirrhosis patients would result in missing about 38% of HCC cases although it should be noted that according to our FIB-4 results about half these patients will be cirrhotic by the time HCC has been detected. Since in most centres, only a minority of patients with CLD will be at the stage of cirrhosis a strategy of monitoring such patients for the development of cirrhosis and subsequent entry into a surveillance programme may need to be considered.

It might be considered that our study, being confined to a Japanese population, may be a limitation but any such validation needs to be undertaken in a population where surveillance is adequately implemented and, as documented elsewhere, this rules out most western countries. The fact that nearly 70% of HCC cases in Ogaki were detected by surveillance and 70% of the HCC cases were detected at a potentially curative stage and actually received potentially curative therapy, attests that this cohort is as close to ideal for HCC surveillance as can be currently contemplated.

With these provisos and with the advantages as described above, our analysis completely confirms the potential of the aMAP score to risk stratify CLD. The model performs well in all aetiologies, irrespective of gender and SVR status, although (similar to the original paper) it performs less well in the cirrhotic cohort and, as we show here, among younger patients. Our conclusions concerning the model performance after SVR should be treated with caution since a considerable period of time may have elapsed between recording the aMAP score on study entry, and achievement of SVR. However, we have recently confirmed the good performance of aMAP in a large series of HCV-cured patients from the UK [20].

Appropriate HCC screening strategies for patients with cirrhosis who have been cured of HCV by the use of DAAs is currently a major clinical concern [21–25] as recently reviewed by Maan et al. [26].

However, we believe that, in the light of our recent demonstration that the serum AFP levels are elevated many years before HCC detection, it is likely that the addition of this simple and widely available test (or the recently developed GALAD score [27, 28]) will improve the performance of aMAP and overcome the present model’s limitations. The difference between the low risk group where there is a 1.3% incidence of HCC and the high risk group where the figure was 37.4% is very striking and the discrimination, as assessed by the ‘*C*’-score was high and almost identical to the original publication. One caveat to note however is that calibration (i.e. agreement between predicted and observed risk) was not optimal, particularly for the highest risk patients where aMAP tended to over-predict the 5-year HCC risk. Thus, as with all prognostic models, aMAP may require recalibration according to geography and time period to ensure the predicted absolute risk of HCC is accurate. Inevitably, there will be ‘loss to follow-up’ in a study spanning >20 years and this may lead to some bias, but, in the event, a figure of >75% for complete follow-up is remarkable [29].

Within any surveillance population, there will be underlying, associated and incidental causes of mortality that have the potential to influence any analysis of the programme. However, we show here that competing causes of death do not have a major impact on the model performance. Furthermore, any disadvantages of our study design are surely outweighed by the ‘immediacy’ of the results. The limitations of formal prospective trials in terms of the long duration required and the changes in clinical practice that occur over the period of the trial is well illustrated by the recently reported UK Collaborative Trial of Ovarian Cancer Screening (UKCTOCS) [30]. This trial involved 200,000 patients and took over 16 years to complete. In the face of negative findings, the authors opined that it would be at least another decade before a further trial could lead to implementation of a practice-changing screening programme.

Our assessment of SMRs in each aMAP class has important implications for practical implementation of HCC surveillance. Identification of subgroups that do not benefit from surveillance and may be excluded from the ‘harms’ related thereto is a realistic possibility. The figure of 1.7 (albeit with wide confidence intervals) as the SMR for the lowest risk group implies a 70% higher risk of death from HCC (than in the general population) and at first sight this may appear very significant but it should be remembered firstly, that some of the increased risk of death will be associated with the attendant CLD which a screening programme could not be expected to affect. Secondly, ‘relative’ figures such as SMR need to be considered in relation to the baseline risk. If the baseline risk to a member of the general population is very low, then a 70% increase may not necessarily be considered unacceptable.

The impact of changes in treatment/management methods introduced over the past 20 years both for the underlying liver disease (nucleoside analogues for HBV, interferon and interferon-free regimens for HCC and general principles such as alcohol avoidance and control of obesity) and the actual HCC (better systemic therapy, for example) can all be investigated within the present cohort. All the additional data required for such model refinement is available and we are currently investigating this possibility. Our analysis suggests the aMAP score possesses all the qualities required for practical risk stratification in HCC surveillance [31].

In conclusion, although it is apparent that certain very low-risk groups are indefinable, the categorisation as low, intermediate and high is arbitrary and the application of aMAP as a continuous variable may be even more revealing. Furthermore, the categorisation of patients as cirrhotic/non-cirrhotic may be simplistic, not allowing for changes in change in degree of fibrosis over time. With these caveats, analysis of this large, prospectively accrued cohort suggest that the aMAP model offers a simple yet robust approach to HCC risk assessment in patients with CLD.

## Supplementary information

Evaluation of the aMAP score for hepatocellular carcinoma surveillance: a realistic opportunity to risk stratify
