# Supplementary material for: Evaluation of the aMAP score for hepatocellular carcinoma surveillance: a realistic opportunity to risk stratify
Source: Br J Cancer. 2022 Jul 7;127(7):1263–9. doi: 10.1038/s41416-022-01851-1 (PMC9519948; doi:10.1038/s41416-022-01851-1)

**SUPPLEMENTARY MATERIAL**

Evaluation of the aMAP score for hepatocellular carcinoma surveillance: a realistic opportunity to risk stratify Johnson, PJ et al

**Table S1.** HCC characteristics at diagnosis

| **Variable** | N=445 |
| --- | --- |
| **Solitary tumour, n (% of group)** | 282 (63.4%) |
| **Tumour size (cm) (Median and range)** | 2  1-12 |
| **Vascular invasion, n (% of group)** | 18 (4.0%) |
| **Within Milan criteria, n (% of group)** | 346 (77.8%) |
| **Subsequent HCC treatment, n (% of group):** |  |
| Potentially curative (all) | 309 (69.4%) |
| Palliative care | 79 (17.8%) |
| Best supportive care | 42 (9.4%) |

**FIGURE S1:** Date of last follow-up for patients not known to have died (N=2729)


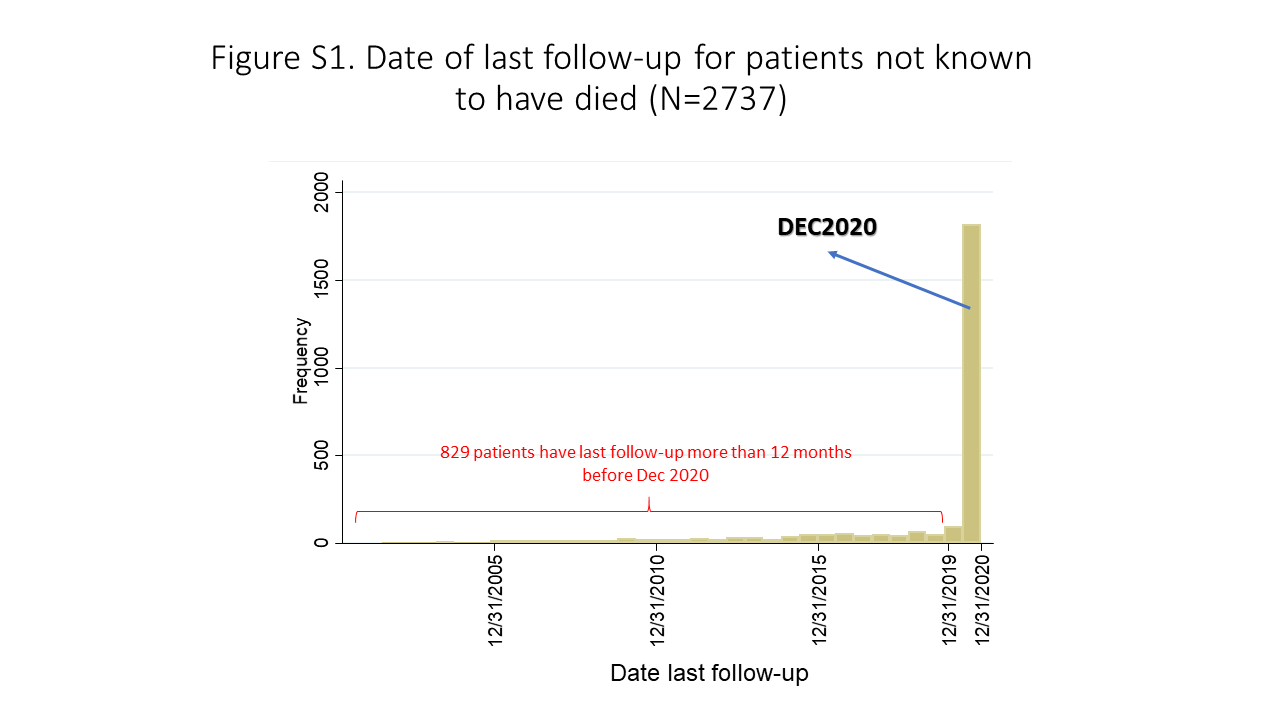


**FIGURE S2:** Discrimination of aMAP score according to time horizon


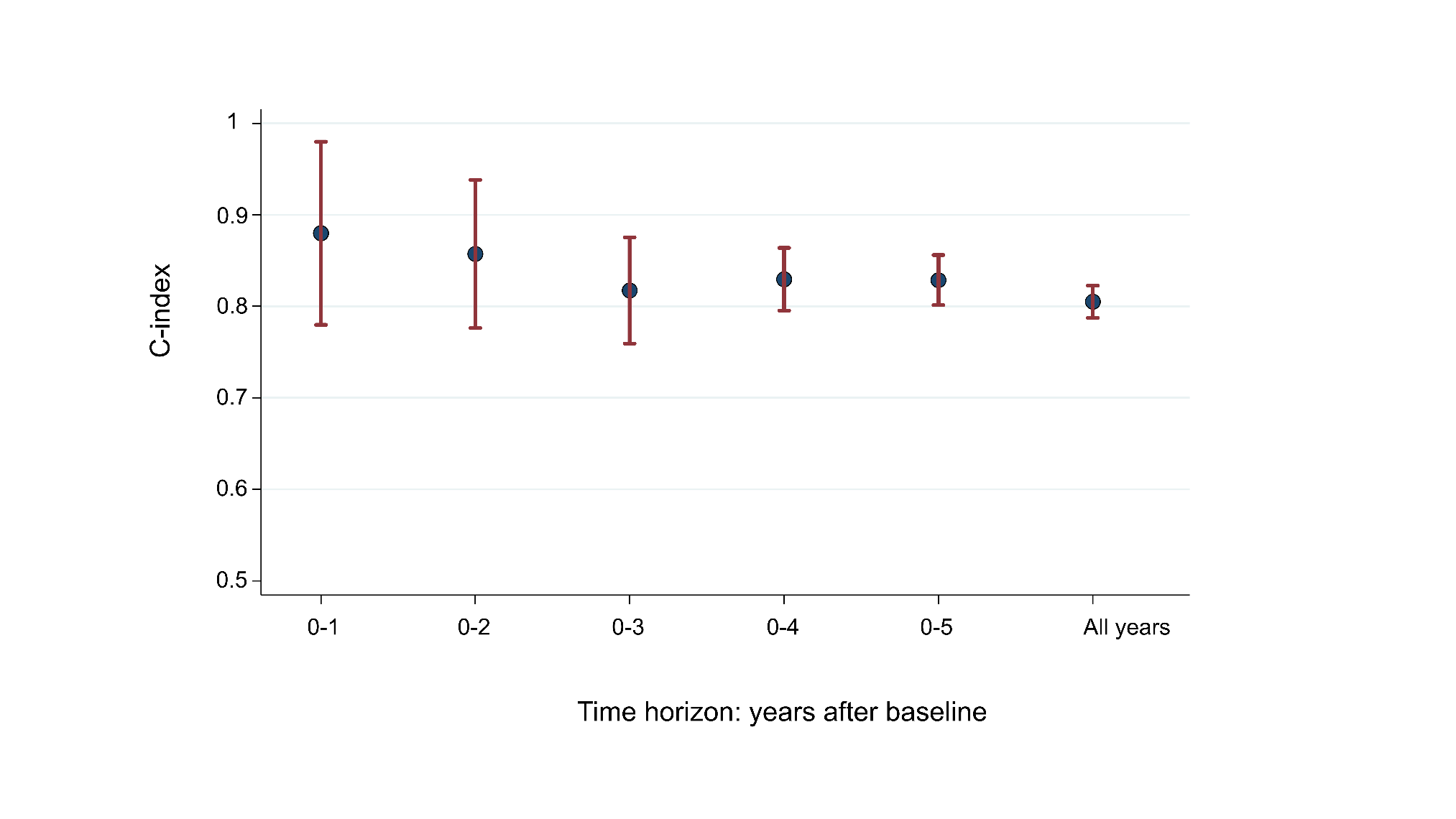


**FIGURE S3:** Calibration plot for the aMAP score in the Ogaki cohort based on 5-year risk prediction. Observed HCC-free probability is defined as 1 minus the cumulative incidence of HCC at five years. The cumulative incidence of HCC differs from the Kaplan Meier estimate of HCC-free survival insofar as it takes into account the competing risk of non-HCC mortality.


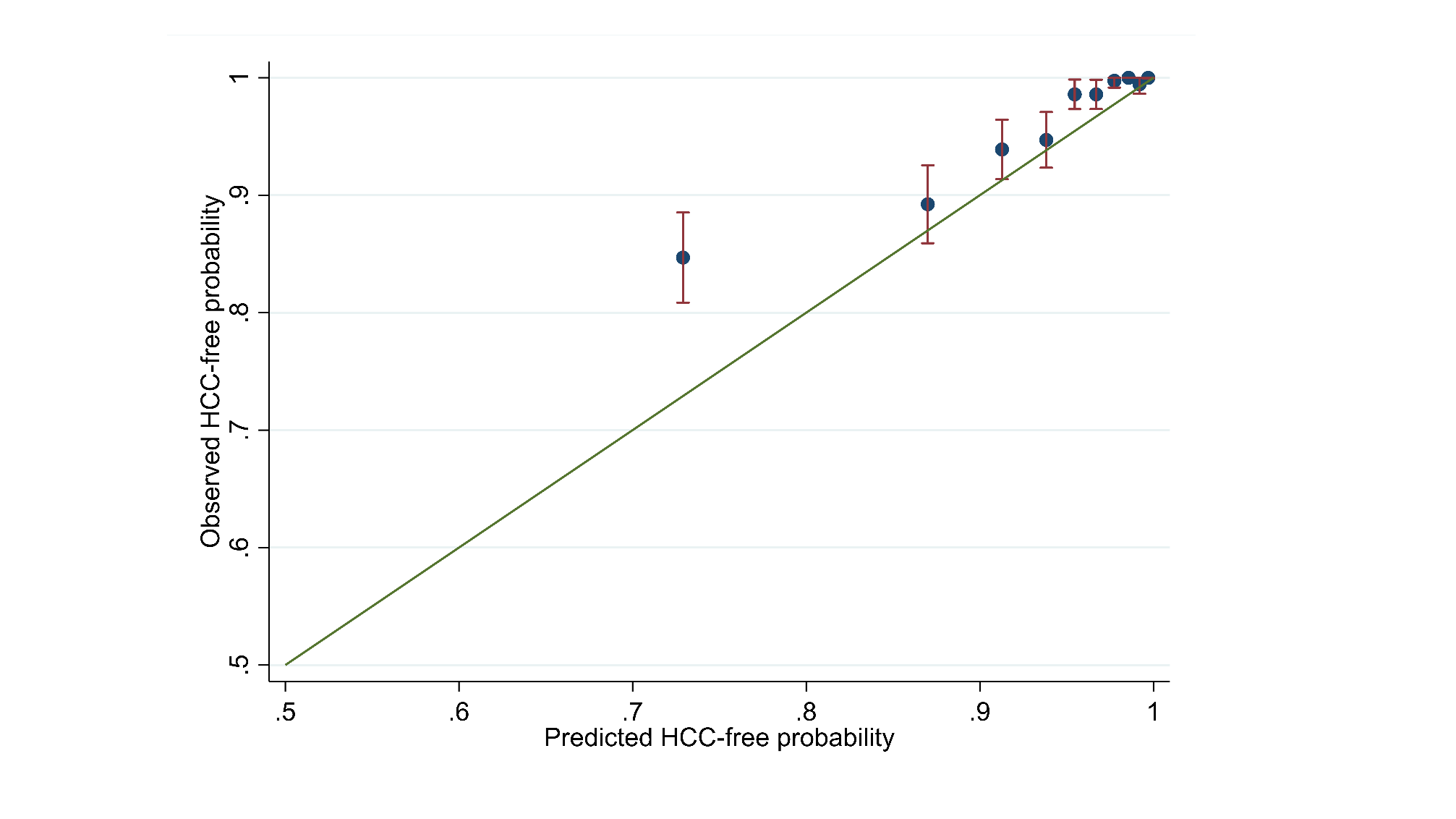

Supplement: Supplementary file 1 — Evaluation of the aMAP score for hepatocellular carcinoma surveillance: a realistic opportunity to risk stratify [file 41416_2022_1851_MOESM1_ESM.docx]
